# Comparison of Effects of Anti-thrombin Aptamers HD1 and HD22 on Aggregation of Human Platelets, Thrombin Generation, Fibrin Formation, and Thrombus Formation Under Flow Conditions

**DOI:** 10.3389/fphar.2019.00068

**Published:** 2019-02-20

**Authors:** Katarzyna Derszniak, Kamil Przyborowski, Karolina Matyjaszczyk, Martijn Moorlag, Bas de Laat, Maria Nowakowska, Stefan Chlopicki

**Affiliations:** ^1^Faculty of Chemistry, Jagiellonian University, Kraków, Poland; ^2^Jagiellonian Centre for Experimental Therapeutics, Jagiellonian University, Kraków, Poland; ^3^Department of Toxicology, Jagiellonian University Medical College, Kraków, Poland; ^4^Cardiovascular Research Institute Maastricht, Maastricht University Medical Centre, Maastricht, Netherlands; ^5^Synapse Research Institute, Maastricht, Netherlands; ^6^Department of Pharmacology, Jagiellonian University Medical College, Kraków, Poland

**Keywords:** aptamers, platelets, thrombin generation, fibrin generation, thrombus formation

## Abstract

HD1 and HD22 are two of the most-studied aptamers binding to thrombin exosite I and exosite, respectively. To complete of their pharmacological profiles, the effects of HD1 and HD22 on thrombin-, ristocetin-, and collagen-induced human platelet aggregation, on thrombin generation and fibrin formation in human plasma, as well as on thrombus formation in human whole blood under flow conditions were assessed. The dissociation constants for HD1 and HD22 complexes with thrombin in simulated plasma ionic buffer were also evaluated. HD1 was more potent than HD22 in terms of inhibiting thrombin-induced platelet aggregation in platelet-rich plasma (PRP; 0.05–3 μM) and in washed platelets (WPs; 0.005–3 μM): approximately 8.31% (±6.99% SD) and 89.53% (±11.38% SD) for HD1 (0.5 μM) and HD22 (0.5 μM), respectively. Neither HD1 nor HD22 (3 μM) did influence platelets aggregation induced by collagen. Both of them inhibited ristocetin-induced aggregation in PRP. Surprisingly, HD1 and HD22 aptamers (3 μM) potentiated ristocetin-induced platelet aggregation in WP. HD1 reduced thrombin generation in a concentration-dependent manner [ETP at 3 μM: 1677.53 ± 55.77 (nM⋅min) vs. control 2271.71 ± 423.66 (nM⋅min)], inhibited fibrin formation (lag time at 3 μM: 33.70 min ± 8.01 min vs. control 7.91 min ± 0.91 min) and reduced thrombus formation under flow conditions [AUC_30_ at 3 μM: 758.30 ± 344.23 (kPa⋅min) vs. control 1553.84 ± 118.03 (kPa⋅min)]. HD22 (3 μM) also delayed thrombin generation but increased the thrombin peak. HD22 (3 μM) shortened the lag time of fibrin generation (5.40 min ± 0.26 min vs. control 7.58 min ± 1.14 min) but did not modify thrombus formation (3, 15 μM). *K*_d_ values for the HD1 complex with thrombin was higher (257.8 ± 15.0 nM) than the *K*_d_ for HD22 (97.6 ± 2.2 nM). In conclusion, HD1 but not HD22 represents a potent anti-thrombotic agent, confirming the major role of exosite I in the action of thrombin. HD22 aptamer blocking exosite II displays weaker anti-platelet and anti-coagulant activity, with surprising activating effects on thrombin and fibrin generation most likely induced by HD22-induced allosteric changes in thrombin dynamic structure.

## Introduction

Thrombin, a central enzyme of the coagulation cascade, plays a crucial role in the formation, growth, and stabilization of thrombi. Thrombin is a highly dynamic molecule involved in the polymerization and stabilization of fibrin, proteolytic and non-proteolytic activation of platelets as well as in the amplification of thrombin generation by feedback activation of upstream coagulation factors. These effects are mostly mediated by thrombin exosite I, exosite II and the active site of the enzyme (Di Cera, 2007; Petrera et al., 2009; Malovichko et al., 2013; Pica et al., 2017).

Given the fact that increased thrombin activity is associated with a variety of cardiovascular diseases (Danckwardt et al., 2013), a number of direct anti-thrombin agents, targeted selectively to thrombin sites, have been developed. For example, bivalent recombinant bivalirudin binding to the active site and exosite I, or univalent direct inhibitors including argatroban and dabigatran binding only to the active site of thrombin (Zavyalova et al., 2016). A number of thrombin binding aptamers (TBAs) have also been recently developed. They represent a promising class of direct anti-thrombin agents with high affinity and specificity to thrombin exosite I or exosite II (Zavyalova et al., 2016) and provide an interesting alternative for clinically used therapeutics (Zhou and Rossi, 2017). The mechanisms of aptamers activity is based on the direct blockage of functionally important sites of thrombin structure resulting in altered function and conformation changes (Petrera et al., 2009). Thus, aptamers are also promising tools for studying the dynamics of the thrombin enzyme molecule.

As regards the use of aptamer as therapeutics, degradation by nucleases and unfavorable pharmacokinetics are limiting factors for their wider therapeutic applications. Various approaches are taken to reduce these limitations (Lakhin et al., 2013; Ni et al., 2017). The use of chemical modification of oligonucleotides including 2′-substitution of the sugar ring by -F, -NH_2_, -OMe, -OH, -H, locked nucleic acid (LNA), unlocked nucleic acid (UNA) or 2′-deoxy-2′-fluoro-D-arabinonucleic acid (2′-F ANA) are only a few examples of solutions to generate nuclease-resistant aptamers. The conjugation of polyethylene glycol (PEG) to aptamers delay their renal filtration (Padilla and Sousa, 1999; Sun et al., 2014; Ni et al., 2017; Maio et al., 2018). The pharmacokinetic parameters of an aptamer could be also controlled by an antidote being a complementary oligonucleotide (Oney et al., 2010; Lakhin et al., 2013; Stoll et al., 2017).

Thrombin binding aptamers include uni- and bivalent oligonucleotides (Müller et al., 2008; Yuminova et al., 2015; Hao and Zhao, 2016; Zavyalova et al., 2016). There is a number of thrombin-binding aptamers including DNA-type NU172, RA-36 binding to exosite I, RNA-type R9D-14T and Toggle-25t binding to exosite I and exosite II, respectively, and DNA-type HD1-22, RNV220, RNV220-T bivalent aptamers (Müller et al., 2008; Yuminova et al., 2015; Hao and Zhao, 2016; Zavyalova et al., 2016). However, HD1 and HD22 are the best known TBAs.

HD1, a 15-mer length oligonucleotide, has a quadruplex structure and binds to thrombin exosite I (Pica et al., 2017), that is positively charged, equipped with hydrophobic patches (Di Cera, 2007), and binds fibrinogen, coagulation factors V, VIII, XI, and XIII, thrombomodulin, heparin cofactor II and the PAR-1 platelet receptor (Verhamme et al., 2002; Bock et al., 2007; Müller et al., 2008). HD1 aptamer inhibits thrombin-induced platelet aggregation by limiting interactions of exosite I with the PAR-1 receptor (Boncler et al., 2001). Because the exosite I region is also present in prothrombin, HD1 inhibits prothrombin activation (Brummel-Ziedins et al., 2005; Kretz et al., 2006, 2010). Despite a potentially interesting profile of HD1 action, it was excluded from further clinical development, because of the lack of expected efficacy and not optimal pharmacokinetic profile (Schwienhorst, 2006; Zavyalova et al., 2017).

HD22, a 29-mer length oligonucleotide, has a duplex-quadruplex structure and interacts with thrombin exosite II (Pica et al., 2017). Positively charged exosite II (Di Cera, 2007) interacts with coagulation factors V and VIII, heparin, prothrombin factor F2 and the platelet GPIb-IX-V receptor (Bock et al., 2007; Müller et al., 2008). HD22 was reported to prolong thrombin-mediated clotting times and to affect interaction of thrombin with fibrinogen (Müller et al., 2008). Interestingly, both aptamers were reported to inhibit non-catalytic fibrin polymerization (Mosesson, 2007) and to suppress coagulation by blocking thrombin-mediated activation of factor V (Segers et al., 2007).

Despite numerous studies, pharmacological effects of HD1 and HD22 are still not fully characterized and some results seem difficult to explain (Tasset et al., 1997; Mosesson, 2007; Müller et al., 2008). There is also a lack of studies in which the mechanisms of their actions are comprehensively compared. Therefore, in the present work, we have compared the pharmacological effects of HD1 and HD22 using quite a comprehensive approach to studying the effects of HD1 and HD22 on platelets, thrombin generation, fibrin formation, and thrombus formation.

We characterized effects of HD1 and HD22 on thrombin-, collagen- and ristocetin-induced platelet aggregation in human washed platelets (WPs) and in human platelet-rich plasma (PRP). We also studied effects of HD1 and HD22 on thrombin and fibrin generation in human platelet-poor plasma (PPP) and on thrombi formation in human whole blood under flow conditions using Total Thrombus-formation Analysis System (T-TAS). Binding characteristics of HD1 and HD22 to exosite I and exosite II was performed in simulated body plasma ionic buffer (SBP) by capillary electrophoresis (CE).

## Materials and Methods

### Blood Collection

Venous blood was obtained from male volunteers at the University Hospital Blood Bank Centre. Volunteer donors had not taken any medicines for the preceding 2 weeks. Informed consent was given by a volunteer prior to the blood withdrawal and study conformed with the principles outlined in the World Medical Association (WMA) Declaration of Helsinki as well as Bioethical Commission of Jagiellonian University.

### Aptamers Preparation

Fluorescein-labeled or non-labeled HD1 (5′-GGTTGGTGTGGTTGG-3′) and HD22 (5′-AGTCCGTGGTAGGGCAGGTTGGGGTGACT-3′) anti-thrombin aptamers (Bock et al., 1992; Tasset et al., 1997) were synthetized by FUTURE Synthesis (Lodz, Poland). Liophilizated anti-thrombin aptamers were dissolved in nuclease-free water (Ambion, United States) and stored at -20°C.

### Light Transmission Aggregometry (LTA)

Prior to measurements, blood samples from healthy volunteers were collected in tubes containing 3.2% sodium citrate (volume ratio: 9:1). Collected blood was centrifuged at 260 × *g* for 15 min at room temperature to obtain PRP and then at 2,600 × *g* for 20 min at room temperature to obtain PPP. PRP was pooled and adjusted to 200,000 platelets/μl with ion-free phosphate-buffered saline (PBS; Lonza, Switzerland) and immediately used for platelet aggregation. PPP was used as blank in LTA carried out in PRP or immediately frozen at -80°C to be tested in thrombin and fibrin generation assays at a later moment.

For the preparation of WPs, PRP was supplemented with prostacyclin (100 ng/ml; Sigma-Aldrich, United States) and centrifuged at 960 g for 10 min. The platelet pellet was re-suspended in ion-free PBS, with addition of prostacyclin (100 ng/ml) and then centrifuged at 810 × *g* for 10 min at room temperature to obtain another platelet pellet. WP were adjusted to 200,000 platelets/μl with ion-free PBS. After a 30-min equilibration period, the platelet suspension was supplemented with CaCl_2_ (Merck, Germany) and MgCl_2_⋅6H_2_O (POCH, Poland) to final concentrations of 9.9 × 10^-4^ mM (Ca^2+^ ions) and 2.1 × 10^-3^ mM (Mg^2+^ ions), respectively, and used for platelet aggregation.

Platelet aggregation was measured by turbidimetry at 37°C under stirring using a Lumi-Aggregometer Model 700 (CHRONO-LOG, United States). For thrombin-induced platelets aggregation tests in PRP, HD1 or HD22 aptamers at final concentrations of 0.0, 0.05, 0.1, 0.3, 0.5, 1.5, and 3 μM were preincubated for 2 min at 37°C in the presence or in the absence of Gly-Pro-Arg-Pro [GPRP, 1.2 mM (Boncler et al., 2001); Sigma-Aldrich, United States], respectively. GPRP suppresses the early stages of fibrin polymerization and was used in platelet aggregation tests assayed in PRP to avoid generation of fibrin. Fibrin fibers formed in plasma during the measurements in Light Transmission Aggregometry (LTA) affect light transmission through cuvettes. The formed fibrin nets intercalate platelets and thrombin molecules which impedes HD1 and HD22 aptamers interactions with these plasma components thus, studied effects could be not only due to the direct effects of TBA on thrombin activity. In the presence of 1.2 mM concentration of GPRP there were no visible fibrin fibers inside the cuvettes. For thrombin-induced platelets aggregation tests in WP, HD1 or HD22 aptamers at final concentrations of 0.005, 0.01, 0.025, 0.05, 0.1, 0.3, 0.5, 1.5, and 3 μM were preincubated for 2 min at 37°C. Thrombin-induced platelets aggregation was also performed in the presence of dabigatran in WP. In this case, dabigatran at 50 ng/ml, corresponding to the therapeutic range of dabigatran peak plasma concentrations (Vinholt et al., 2017), was preincubated for 2 min at 37°C. For ristocetin- and collagen-induced platelets aggregation tests in PRP and WP, HD1 or HD22 aptamers at final concentration of 3 μM were preincubated for 2 min at 37°C. Then, platelets were stimulated with natural human thrombin (0.5 U/ml; Abcam, GB), ristocetin (PRP: 0.75 mg/ml; WP: 1 mg/ml; Biogenet, Poland) or collagen (2 μg/ml; Biogenet, Poland). Platelet aggregation was monitored for 6 min and results were presented as a percent of control aggregation for thrombin- and collagen-induced aggregation tests and as platelets aggregation expressed as a percentage for ristocetin-induced platelet aggregation.

### Calibrated Automated Thrombography (CAT)

Immediately prior to measurements, frozen PPP plasma samples were thawed at 37°C. Thrombin generation in control samples was activated by mixing 40 μl plasma with 10 μl of fluorogenic substrate (Z-Gly-Gly-Arg-AMC, 16.6 mM; Thrombinoscope B.V., Netherlands) and 10 μl of trigger solution containing phospholipids (PL, 4 μM), tissue factor (TF, 1 pM) (Thrombinoscope B.V., Netherlands) and CaCl_2_ (16.6 Mm). HD1 or HD22 aptamers were added to reach the final concentrations of 0.5, 3 and 15 μM. In the calibration wells, the 10 μl of reagents were replaced with calibrator (complex of alpha 2 macroglobulin with thrombin, α_2_M-T, 208 nM; Thrombinoscope B.V., Netherlands). Immediately after the activation of thrombin generation, 60 μl of the mixture was pipetted into a flat-bottom 96-well polystyrene plate. Fluorescence signals were measured using a plate reader (λ = 390 nm, λ = 460 nm; Spark Magellan^®^, Tecan, Switzerland) and transformed into thrombin concentration as described previously (Hemker et al., 2003; Hemker and Kremers, 2013).

Furthermore, the effect of HD1 and HD22 aptamers on thrombin activity toward fluorogenic substrate was investigated. Fluorescence signal generation was triggered by adding 10 μl BSA60 buffer (20 mM Hepes, 6% BSA, 0.02% NaN_3_; pH = 7.35; Thrombinoscope B.V., Netherlands) containing fluorogenic substrate (Z-Gly-Gly-Arg-AMC, 16.6 mM) to 50 μl of BSA60 buffer supplemented with human thrombin (1 U/ml) and HD1 or HD22 aptamers (3 μM). The reference systems were prepared as above but without the aptamers. The fluorescence signal was recorded using the plate reader (λ = 390 nm, λ = 460 nm; Spark Magellan^®^, Tecan, Switzerland).

### Turbidimetry Measurements

Fibrin generation was triggered by addition of PPP to the wells of the microtiter plate containing TF (0.5 pM), PL (4 μM) and calcium ions (Ca^2+^, 10 mM) in the presence or absence of HD1 or HD22 aptamers (3 μM). After mixing of PPP with reagents, the turbidity of the solution was continuously monitored by measuring absorbance using the plate reader (λ = 405 nm; Spark Magellan^®^, Tecan, Switzerland) at 37°C. The influence of HD1 and HD22 aptamers on fibrin generation was evaluated in nine (*n* = 9) independent measurements. *T*_1/2_ values were calculated as time period that elapsed to obtain the half of maximal plateau absorbance and was presented as mean ± SD (*n* = 9).

### Total Thrombus-Formation Analysis System (T-TAS)

Citrated whole blood was preincubated (2 min, room temperature) with HD1 or HD22 anti-thrombin aptamers (3 μM or 15 μM). Samples were mixed with a CaCl_2_ (12 Mm) solution containing corn trypsin inhibitor (CTI, 50 μg/ml; Zacros, Japan). After mixing, each blood sample was immediately perfused over the microchip with thrombogenic surfaces (collagen and tissue factor, atheroma AR chip; T-TAS; Fujimori Kogyo Co., Ltd., Japan) at a flow rate of 4 μl/min. Flow pressure changes were monitored by the pressure transducer located upstream of the microcapillaries. The pressure pattern for each sample was used to analyze thrombus formation based on the following estimated parameters: time required to reach 10 kPa from baseline pressure, reflecting onset time for thrombus formation (T10; min); occlusion time (OT; min), which is the time reflecting complete capillary occlusion and area under a time vs. pressure curve (AUC_30_) obtained after 30 min (under 80 kPa) since the start of the assay, reflecting total thrombogenicity.

Thrombus formation inside capillaries was recorded in real time using a T-TAS CCD camera at 0, 3, 6, 9, and 12 min after blood sample application and the start of the measurement. The two-dimensional area covered by thrombi was analyzed by image analysis software (Zia 4.0.0.1, Fujimori Kogyo Co., Ltd., Japan).

### Capillary Electrophoresis (CE)

A P/ACE MDQ capillary electrophoresis system (Beckman Coulter, Fullerton, CA, United States) with a Laser-Induced Fluorescence (LIF) detector equipped with an air-cooled argon laser (Beckman Instruments, United States) with excitation at 488 nm and emission at 520 nm was used.

Simulated body plasma ionic buffer (SBP) was used as the incubation buffer, prepared according to the European Standard EN ISO 10993-15:2009 [NaCl (0.116 M), CaCl_2_ (0.002 M), KCl (0.005 M), NaHCO_3_ (0.026 M) (Merck, Germany), MgSO_4_ (0.001 M), Na_2_HPO_4_⋅2H_2_O (0.001 M) and NaH_2_PO_4_ (0.00017 M) (Sigma-Aldrich, United States)].

To evaluate interactions of aptamers with human thrombin, the experiments were performed using a bare fused silica capillary (BFS; 30.2 cm total length, 20 cm effective length, 50 μm i.d., 375 μm o.d., thermostated at 25°C). Prior to each run, the BFS capillary was rinsed with 0.1 M NaOH (Sigma-Aldrich, United States), followed by H_2_O and running buffer (275.8 kPa, 2 min for each). After sample injection (hydrodynamic injection ∼20 nl; capillary pressure pulse: 3.4 kPa, 10 s) separation was performed using buffer (pH = 8.3) containing 20 mM TRIS and 9 mM acetic acid (Sigma-Aldrich, United States). A voltage of +10 kV, which produced an electric field of approximately 333 V⋅cm^-1^, was used.

Fluorescein-labeled aptamers were used for evaluation of interactions of aptamers with thrombin. Prior to analyses, aptamers were heated to 95°C (5 min) and then slowly cooled to room temperature. Samples contained 400 nM thrombin and 100 nM aptamer prepared in SBP were incubated at 37°C for 2 min before measurements. Control samples contained 100 nM of each aptamer.

### Calculations of Dissociation Constants (*K*_d_)

*K*_d_ calculations were based on the non-equilibrium capillary electrophoresis of equilibrium mixtures (NECEEM) method introduced by Krylov (2006). Briefly, a short plug of the equilibrium mixture (consisting of ligand, its target and complex which is created during incubation) is injected into the inlet of the capillary. Separation is carried out with the running buffer only. The complex continuously dissociates during electrophoresis. If separation is efficient, reassociation of target and ligand can be neglected. Consequently, the characteristic electropherograms are obtained, which contain peaks from free ligand, target and complex – and two exponential ‘smears’ of ligand and target unleashed during separation.

### Statistical Calculations

Values are presented as mean ± SD. Normal distribution of data was verified by Shapiro–Wilk test. Statistical significance of differences between groups was estimated by an unpaired *t*-test (ristocetin-induced platelet aggregation in PRP for HD1 and in WP for HD1 and HD22), Mann–Whitney test (ristocetin-induced platelet aggregation in PRP for HD22 and T-TAS measurements), a non-parametric Kruskal–Wallis test followed by *post hoc* multiple comparisons Dunn’s test (for CAT measurements). A *p*-value < 0.05 was considered statistically significant (^∗^*p* < 0.05, ^∗∗^*p* < 0.01, ^∗∗∗^*p* < 0.001, ^∗∗∗∗^*p* < 0.0001). Analyses were performed using GaphPad Prism 6.0 software. The power of the test was verified by a *post hoc T*-test of differences between two independent means (α = 0.05). Analysis of the test power was performed by G^∗^ Power 3.1.9.2 software.

## Results

### Effects of HD1 and HD22 Aptamers on Thrombin-, Collagen-, and Ristocetin-Induced Platelet Aggregation in Washed Platelets and in Platelet-Rich Plasma

**FIGURE 1:**
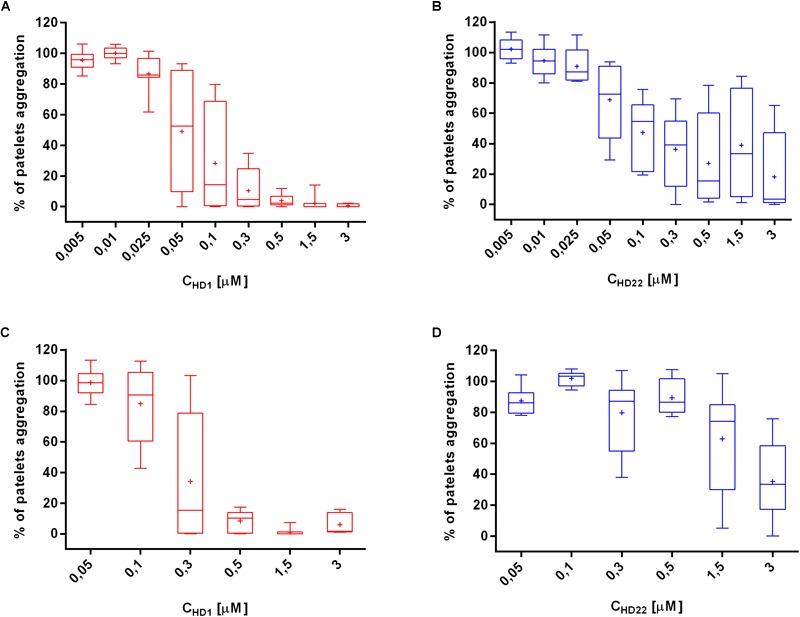
Effects of HD1 and HD22 aptamers on thrombin-induced platelet aggregation in WP **(A,B)** and in PRP **(C,D)**. Data distribution is presented as median with box plots and 25th and 75th percentile. Data mean is represented as a cross (*n* = 8).

HD1 strongly inhibited thrombin-induced platelet aggregation in a concentration-dependent manner (0.05–3.0 μM) in PRP (Figure 1C). HD1 at 3 μM concentration caused total inhibition of platelets aggregation in PRP, while HD22 (3 μM) inhibited platelets aggregation to 35.22% of control (Figure 1D). At lower concentrations, HD1 (0.5 μM) was about ten times more potent than HD22 (0.5 μM) in terms of inhibiting thrombin-induced platelet aggregation in PRP (8.31% ± 6.99% vs. 89.53% ± 11.38%, respectively). The anti-thrombin effect of HD1 (0.005–3 μM) was even stronger in WPs (Figure 1A), but the difference between potency of HD1 and HD22 (Figure 1B) in WP was smaller than in PRP.

**FIGURE 2:**
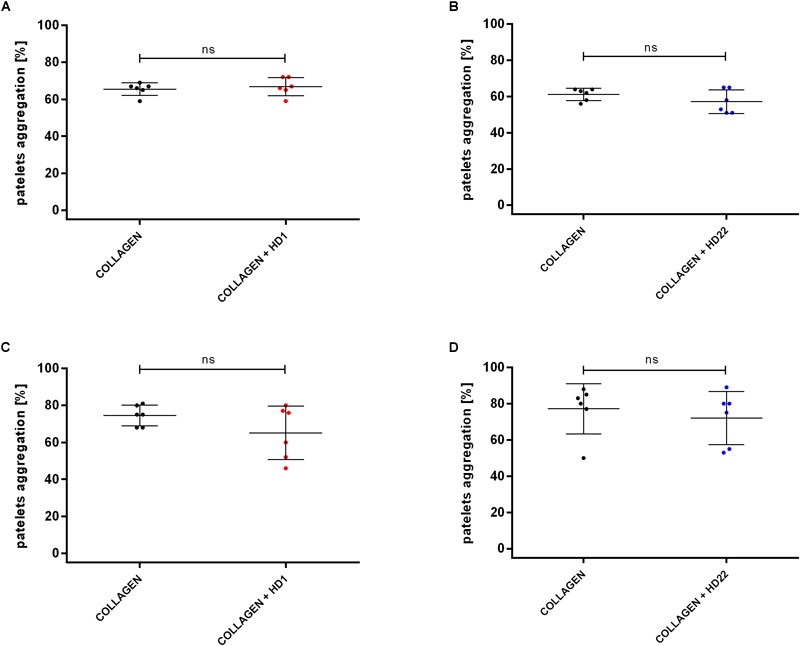
Effects of HD1 and HD22 aptamers (3 μM) on collagen-induced (2 μg/ml) platelet aggregation in WP **(A,B)** and in PRP **(C,D)**. Results are presented as mean ± SD.

HD1 and HD22 aptamers (3 μM) did not affect collagen-induced platelet aggregation in PRP and WP (Figure 2A–D).

**FIGURE 3:**
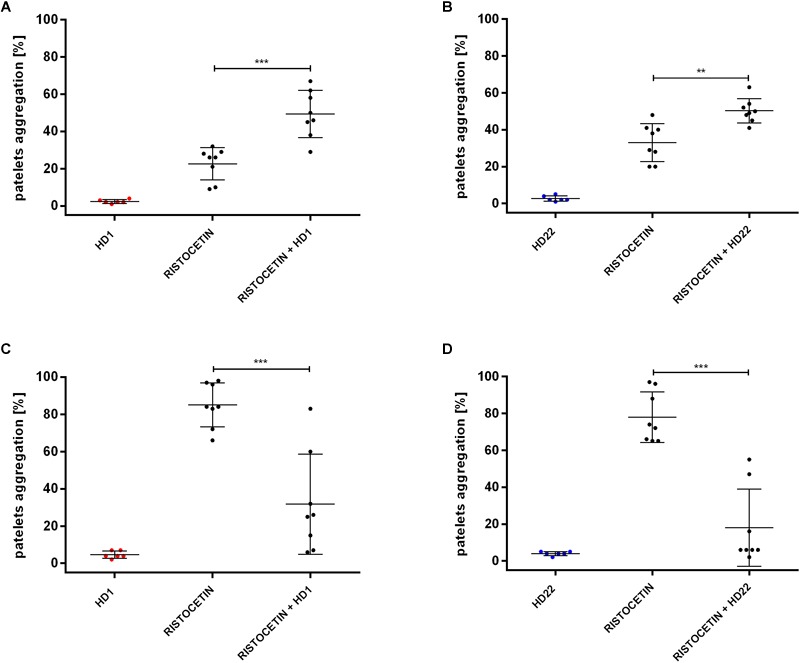
Effects of HD1 and HD22 aptamers (3 μM) on ristocetin-induced platelet aggregation in WP (**A,B**; ristocetin at 1 mg/ml) and in PRP (**C,D**; ristocetin at 0.75 mg/ml). Results are presented as mean ± SD.

HD1 and HD22 aptamers (3 μM) caused statistically significant inhibition of ristocetin-induced platelet aggregation (to 31.75% ± 26.88% and 18.00% ± 20.86% of control in PRP (Figure 3C,D).

However, in WP, both HD1 and HD22 potentiated the ristocetin-induced platelets aggregation (Figure 3A,B). The percentage of platelets aggregation in WP was 49.38% (±12.65% SD) or 50.25% (±6.54% SD) in the presence of HD1 or HD22 aptamer in contrast to control measurements of 22.63% (±8.68% SD) or 33% (±10.30% SD), respectively.

For comparison, we assessed effects if dabigatran at 50 ng/ml (0.11 μM), which corresponds to the therapeutic range of dabigatran peak plasma concentrations (Vinholt et al., 2017), that resulted in total inhibition of platelets aggregation caused by thrombin in WP (0% ± 0% vs. 79.40% ± 3.65% control, *n* = 5).

### Effects of HD1 and HD22 Aptamers on Thrombin Generation in Human Platelet-Poor Plasma

**FIGURE 4:**
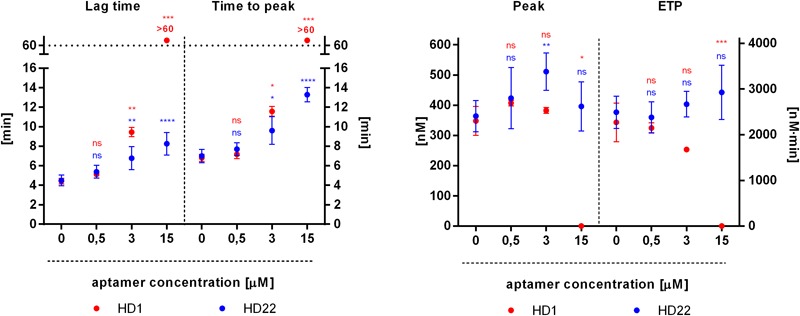
Effects of HD1 and HD22 aptamers on thrombin generation assessed by calibrated automated thrombography (CAT) in human platelet poor plasma. Results are presented as mean ± SD (*n*_HD1_ = 4, control of HD1 *n* = 12; *n*_HD22_ = 6, control of HD22 *n* = 15).

HD1 aptamer inhibited thrombin generation in a concentration-dependent manner, as evidenced by a prolongation of the lag time and the time to peak, as well as lowered values of peak and endogenous thrombin potential (ETP) (Figure 4). HD1 at a concentration of 0.5 μM was not effective while, at 3 μM HD1, it significantly prolonged the lag time (from 4 to 10 min), the time to peak (from 7 to 12 min) and decreased the ETP (from 2.270 to 1.670 nM⋅min) as compared to control (Figure 4). HD1 aptamer at a concentration of 15 μM completely inhibited thrombin generation, as evidenced by the readout of all four parameters (Figure 4). Effects of HD22 aptamer on thrombin generation were weaker as compared with HD1. However, HD22 still prolonged the lag time and the time to peak in a concentration-dependent manner, with about a twofold increase of these parameters at a concentration of 15 μM (for the lag time from 4 to 8 min and the time to peak from 7 to 13 min). Unlike HD1, HD22 was not reduced but it rather tended to increase the height of the peak, particularly at the concentration of 3 μM (from 364 nM to 511 nM), and the ETP at the concentration of 3 μM and 15 μM (from 2.490 nM⋅min to 2.667 nM⋅min and to 2.927 nM⋅min, respectively) in comparison to control.

HD22 enhanced thrombin catalytic activity toward the CAT fluorogenic substrate. The fluorescence signal reached 4952 A.U. after 60 min of measurement, in comparison with 3108 A.U. of the control signal. In contrast, HD1 did not have an influence on fluorescence generation. The fluorescence signal reached 3403 A.U. after 60 min of measurement in comparison to 3316 A.U. of the control signal.

### Effect of HD1 and HD22 Aptamers on Fibrin Formation in Platelet-Poor Plasma

**FIGURE 5:**
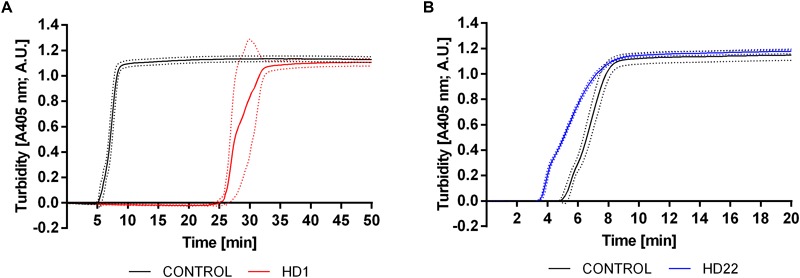
Representative curves showing the effect of HD1 (3 μM) **(A)** and HD22 (3 μM) **(B)** on fibrin formation in human platelet poor plasma evaluated by turbidimetry measurements. Results are presented as mean (solid line) ± SD (dotted line) obtained from four independent measurements.

HD1 aptamer at a concentration of 3 μM prolonged the lag time by approximately 20 min in comparison to control (Figure 5A). HD22 aptamer at the same concentration demonstrated the opposite effect and shortened the lag time by approximately 1.5 min in comparison to control (Figure 5B). Calculated *t*_1/2_ values were approximately 33.70 min ± 8.01 min and 5.40 min ± 0.26 min for HD1 and HD22, respectively, in comparison to respective controls (7.91 min ± 0.91 min and 7.58 min ± 1.14 min, *p* < 0.0001). HD22 activity did not influence the maximal absorbance (1.12 A.U.), which was close to the control value (1.11 A.U.). A slight reduction of the amount of generated fibrin was obtained for HD1, where the maximal absorbance was 0.95 A.U. compared to 1.09 A.U. for the control.

### Effects of HD1 and HD22 on Thrombus Formation in Human Whole Blood

**FIGURE 6:**
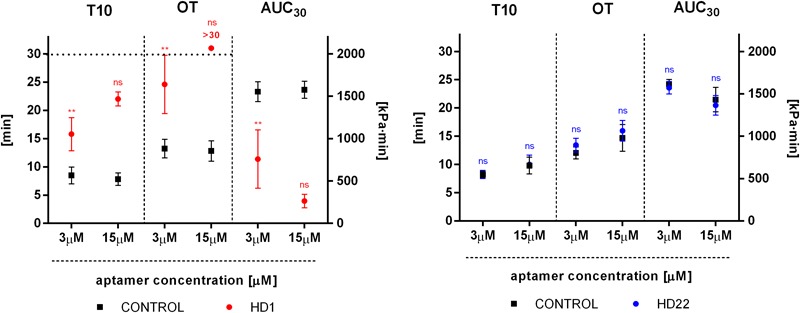
Effect of HD1 and HD22 aptamers on thrombus formation in human blood in a microchip-based flow chamber system (Total Thrombus-formation Analysis System, T-TAS). T10, thrombi formation starting time; OT, occlusion time; AUC_30_, area under time-pressure curve. Results are presented as mean ± SD (3 μM: *n*_HD1_ = 5, *n*_HD22_ = 3; 15 μM: *n*_HD1_ = 3, *n*_HD22_ = 5). *Post hoc* compute test power achieved *p* = 1.00 for all parameters (T10, OT, AUC_30_) for 15 μM of the HD1 aptamer.

**FIGURE 7:**
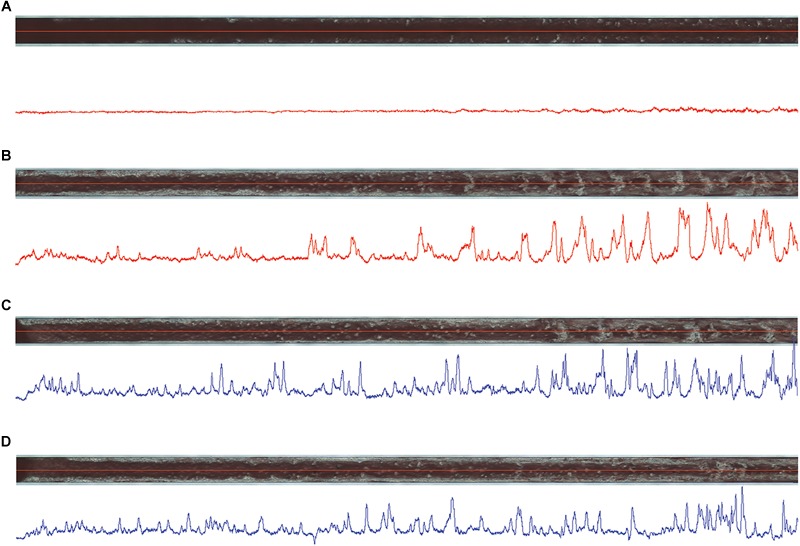
Images of thrombi formed inside capillaries of atheroma chips (AR) under flow conditions in HD1- **(A)** and HD22-treated **(C)** (both at 15 μM) blood samples obtained from one volunteer in comparison to controls **(B,D)**, respectively, at 12 min of measurements. Blue and red lines below the images correspond to thrombi profile in the middle of capillaries, where high peaks correspond to high density of thrombi inside capillaries.

HD1 inhibited thrombus formation in a microchip-based chamber system (T-TAS) in a concentration-dependent manner (3-15 μM), as evidenced by the delay of the start time of thrombi formation (T10), the prolongation of the occlusion time (OT) and reduction of total thrombogenicity (AUC_30_) (Figure 6). HD1 at a concentration of 15 μM substantially prolonged the occlusion time up to more than 30 min. Additionally, fibrin formation was profoundly reduced by the HD1 aptamer, as evidenced by observations of images inside capillaries recorded by a T-TAS CCD camera (Figure 7). HD1 aptamer at a concentration of 15 μM significantly decreased the area covered by thrombi in microcapillaries (3,6%) as compared to the corresponding control sample (33.1%) measured at 12 min after blood sample application. In contrast, HD22 had no impact on thrombus formation at any studied concentrations (3–15 μM), as shown by unchanged values of T10, OT and AUC_30_ (Figure 6). For a 15 μM concentration of HD22 aptamer, the area inside the chip covered by thrombi was 33.6%, which was similar to the control value (31.2%) (Figure 7).

### HD1 and HD22 Affinity to Human Thrombin in Simulated Body Plasma Ionic Buffer

As shown in Table 1, HD22 aptamer affinity to thrombin was higher than that of HD1 as measured by capillary electrophoresis in simulated body plasma ionic buffer (SBP).

**Table 1:** Representative values of dissociation constants of HD1 and HD22 aptamer complexes with human thrombin measured in simulated body plasma ionic buffer.

Incubation buffer	*K*_d_^HD1^± SD (nM)	*K*_d_^HD22^± SD (nM)
SBP	257.8 ± 15	97.6 ± 2.2

*K_*d*_, dissociation constants; SBP, simulated body plasma ionic buffer. Means and standard deviation (SD) are presented.*

## Discussion

In the present work, using a comprehensive approach, we have compared the pharmacological effects of two thrombin binding aptamers, HD1 and HD22. We characterized their activity toward platelets and thrombin-dependent coagulation processes in various assays including thrombin-, collagen-, and ristocetin-induced platelet aggregation in human PRP and in WPs, thrombin and fibrin generation in human PPP as well as thrombus formation in human blood under flow conditions encompassing platelets and coagulation cascade activity together using a microchip-based system (T-TAS).

We demonstrated that HD1 is a potent anti-platelet, anti-coagulant, and anti-thrombotic agent, underscoring the major role of exosite I in the modulation of thrombin action. In turn, although HD22 (an exosite II biding aptamer) had a higher affinity to bind thrombin as evidenced by lower *K*_d_ values, it displayed weaker anti-platelet activity in WP and PRP as compared with that of HD1, displayed a weaker anti-coagulant activity, with a surprising activating effects on thrombin and fibrin generation most likely induced by HD22-induced allosteric changes in thrombin dynamic structure. HD22 did not display anti-thrombotic nor pro-thrombotic effect on thrombus formation in human blood under flow conditions.

In thrombin-induced platelet aggregation experiments, HD1 aptamer was a stronger anti-platelet agent than HD22 in PRP as well as in WP (Figure 1A–D). Differences in the strength of anti-platelet effects of HD1 and HD22 aptamers might be explained considering the interaction of thrombin exosite I and exosite II with platelet receptors. Exosite I interacts with the PAR-1 receptor (Pica et al., 2017), representing a dominant membrane component of thrombin-induced proteolytic platelet activation mechanism (Nieman, 2016). HD1 aptamer blocks the initiation of platelet aggregation preceded by proteolytic cleavage of the PAR-1 receptor (Brien et al., 2001).

On the other hand, effects of the HD22 aptamer on platelet aggregation reported here seem to be compatible with a role of thrombin exosite II (Pica et al., 2017) in activation of the GPIb-IX-V receptor, representing an additional mechanism of thrombin-dependent platelet activation different than proteolytical activation of PAR-1, the mechanism of which is still not clear (De Candia et al., 2001; De Candia, 2012). Weak anti-platelets effects of HD22 might be also link to the blockage of exosite II which leads to slight inhibition of hydrolysis of PAR-1 receptor following the action of exosite I (De Candia et al., 2001; De Candia, 2012). Allosteric changes and interdependences between exosite II, exosite I and the active site of thrombin which could cause the reduction of strength of interactions of PAR-1 with exosite I should also be considered (Petrera et al., 2009).

Additionally, HD1 and HD22 showed non-specific interactions under the absence of thrombin resulting in the inhibition and enhancement of ristocetin-induced platelet activation in PRP and WP, respectively (Figure 3A–D). Aggregation of platelets evoked by ristocetin is dependent on conformational changes of von Willebrand factor facilitating the protein interaction with GPIb-IX-V receptor (Kang et al., 2008; Obert et al., 2014) and that is why, ristocetin-induced platelet aggregation (RIPA) test was used to a diagnosis of von Willebrand disease (Horvath et al., 2004; Ruggeri, 2007; Castaman et al., 2014).

Here was shown that HD1 and HD22 reduced ristocetin-induced platelet aggregation in PRP (Figure 3C,D) suggesting that HD1 and HD22 aptamers bind to GPIb-IX-V receptor impeding interactions of soluble vWF with the receptor resulting in the inhibition of the ristocetin-induced response of platelets in the presence of plasma-derived vWF known to interact with GPIb-IX-V receptor, in contrast to platelet-derived vWF interacting rather with GPIIb-IIIa receptor (Gralnick et al., 1991).

However, in WP, both HD1 and HD22 increased platelet activation, most likely by direct interactions of HD1 and HD22 with GPIb-IX-V receptor leading to the release of vWF from platelets α-granules which caused platelet aggregation mediated by binding of secreted vWF to GPIIb-IIIa receptor in WPs samples. The α-granules-derived vWF accounts only about 20% of total protein in blood (Peyvandi et al., 2011; Mcgrath et al., 2018; Verhenne et al., 2018) which means that the involvement of α-granules-derived vWF in HD1 and HD22 effects in PRP or in full blood might be negligible. However, it might well be that this additional mechanism of HD1 and HD22 aptamers actions could explain the presence of significance variabilities of obtained results especially among platelets aggregation data and the need of use of high doses of HD1 and HD22 in *ex vivo* T-TAS assay.

In contrast to a possible interactions of HD1 and HD22 aptamers with GPIb-IX-V receptor neither of the aptamers did influenced collagen-induced platelet aggregation excluding the interactions of HD1 and HD22 with GPVI receptor (Miura et al., 2002; Nieswandt and Watson, 2003).

In the present work, we compared effects of thrombin binding aptamers with that of dabigatran (50 ng/ml) that clearly show that effects of most effective aptamer HD1 on thrombin-induced platelet aggregation was weaker than dabigatran used in the therapeutic range of concentration, underscoring weak therapeutic potential of HD1 as reported in clinical trials (Mayer et al., 2011; Vinholt et al., 2017; Zavyalova et al., 2017).

Independently of effects on platelets, HD22 (3 μM) but not HD1 (3 μM), surprisingly displayed enhanced fluorogenic signal production, assessed by calibrated automated thrombography (CAT), an increasingly appreciated reference method for the quantitative assessment of thrombin generation (Castoldi and Rosing, 2011; Hemker and Kremers, 2013). Apart from the weak anticoagulant effects of the HD22 aptamer, as evidenced by a prolonged lag time and time to peak, HD22 increased the height of the peak and ETP (Figure 4). We also observed a higher fluorescence signal in measurements of fluorogenic signal generation during CAT fluorogenic substrate cleavage by thrombin in the presence of HD22 aptamer (3 μM) as compared to control in buffer matrix while HD1 aptamer (3 μM) did not show any effect. We hypothesized that these surprising results of HD22 could be explained by the conformational changes of exosite I and/or the active site of the thrombin caused by binding of HD22 to exosite II, resulting in the limited coagulation factors activation and enhancement of the catalytic activity of thrombin toward the fluorogenic substrate (Fredenburgh et al., 1997; Bock et al., 2007; Segers et al., 2007).

Similar effect was seen when, instead of CAT substrate, fibrinogenesis was measured. We observed the acceleration of fibrin formation by HD22 (3 μM) (Figure 5B) as evidenced by shortened lag time and reduced *t*_1/2_ value as compared to control, which supports the notion of HD22 interaction with exosite II, induce changes in the conformation of thrombin. We hypothesize that the active site of thrombin might be allosterically changed toward acceleration of fibrinogen cleavage resulting in accelerated fibrin generation and/or the affinity of thrombin exosite I to fibrinogen may increase.

In contrast to the complex effects of HD22 on thrombin and fibrin generation, HD1 inhibited thrombin generation in a concentration-dependent manner (0.5–15 μM) and fibrin generation (3 μM), as evidenced by prolongation of lag time, time to peak, and lowered endogenous thrombin potential (ETP) in CAT measurements (Figure 4) and prolonged lag time and *t*_1/2_ parameter (Figure 5A) as compared to controls in turbidity measurements, which all support the major role of exosite I in the activity of thrombin (Pospisil et al., 2003; Kretz et al., 2006; Crawley et al., 2007).

Altogether, comparison of the effects of HD1 and HD22 on thrombin generation and fibrin formation revealed strong and clear-cut anti-coagulant effects of HD1 aptamer and modest and more complex mechanisms of actions of HD22 aptamer on coagulation and fibrin generation, underscoring the dynamic nature of thrombin (Petrera et al., 2009; Malovichko et al., 2013; Pica et al., 2017).

For the final readout of the thrombotic effects of HD1 and HD22, we used the novel microchip-based chamber system for thrombi formation in human whole blood (*ex vivo*) under flow conditions (T-TAS). Using microchips covered by collagen and tissue factor we compared effects of both aptamers. This experimental approach demonstrated that HD1 (3 μM, 15 μM) displayed concentration-dependent anti-thrombotic effects, as reflected by the delayed start of thrombus formation (T10), prolonged occlusion time (OT) and reduced total thrombogenicity (AUC_30_) (Figure 6) that was associated with the reduction of the area covered by thrombi by approximately 30% in comparison with the control samples (Figure 7). These results indicated that HD1 aptamer effectively limited the interactions of fibrinogen and platelets receptors with exosite I of thrombin as well as thrombin generation and coagulation process.

In contrast to HD1, HD22 (3 μM, 15 μM) did not influence significantly any measured parameters (Figure 6, 7). The thrombogenic surface of AR chips, which were used during experiments, induced robust thrombin production and platelet activation. Even though blood samples are rich in soluble von Willebrand factor which interacts fervently with platelet receptor GPIb-IX-V and collagen under shear stress conditions (Gralnick et al., 1991; Siedlecki et al., 1996; Andrews et al., 2003). Collagen surfaces may also strongly activate platelets directly by the GPVI receptor (Farndale, 2006).

Altogether, the weak anti-platelet effects of HD22 observed in PRP or WP that we attributed to the GPIb-IX-V receptor, and its weak anti-coagulation effects observed in CAT as well as the effects of HD22 in non-catalytic fibrin polymerization (Mosesson, 2007), are apparently of negligible importance in thrombi formation in human blood inside AR chips, whereby mechanisms of thrombin-mediated fibrin formation, platelet activation and adhesion triggered by collagen, and TF surface, are dominant.

To correlate pharmacological activity of HD1 and HD22 aptamers with their affinity to thrombin, we determined *K*_d_ values for both using capillary electrophoresis (CE) in simulated body plasma ionic buffer (SBP). Pica et al. showed a sandwich complex of HD1 and HD22 with thrombin (Pica et al., 2017). However, the influence of matrix composition on conformation and complex stabilization have not been fully elucidated. Measurements of aptamer affinity to thrombin prepared in SBP showed stronger interaction of HD22 than HD1 aptamer with thrombin. *K*_d_ values obtained were equal to 97.6 nM and 257.8 nM for HD22 and HD1, respectively (Table 1). That difference can be related to the longer oligonucleotide strain of HD22 aptamer as compared to the length of the HD1 strain (Spiridonova et al., 2015) but was not translated to better anti-thrombin activity of HD22 as compared with HD1, due to a quite different role of exosite I vs. exosite II in the regulation of thrombin activity.

## Conclusion

To conclude, HD1 but not HD22 can be considered as a potent anti-thrombin agent. This result is compatible with the major role of exosite I in platelet activation, thrombin generation, fibrin generation and thrombus formation. The exosite I interactions with PAR1, fibrinogen and coagulation factors could all be involved (Verhamme et al., 2002; Bock et al., 2007). HD22 aptamer interactions with thrombin at exosite II displays weak anti-platelet and procoagulant effects, without any evident anti-thrombotic effect, underscoring the complex nature of exosite II-dependent regulation of thrombin function, including direct interactions of the aptamer with exosite II as well as allosteric interactions inside the enzyme molecule. Finally, our results demonstrated non-specific, thrombin-independent mechanisms of HD1 and HD22 action most likely linked to their interactions with GPIb-IX-V receptor. Altogether, our results seem to question the superiority of bivalent anti-thrombin aptamer binding to exosite I and exosite II over those binding to exosite I only, as has been recently proposed (Müller et al., 2008; Hughes et al., 2017).

## Author Contributions

KD, KP, and SC conceived and designed the research. KD, KP, and KM carried out the experiments. MM and BL contributed with analytic tools. KD, KP, KM, and MM performed the data analysis. KD and SC drafted the manuscript and wrote the final version of the manuscript. KD, KP, KM, MM, BdL, and MN revised the manuscript. All authors read and approved the final manuscript.

## Conflict of Interest Statement

The authors declare that the research was conducted in the absence of any commercial or financial relationships that could be construed as a potential conflict of interest.
